# Preparation of ractopamine-tetraphenylborate complexed nanoparticles used as sensors to rapidly determine ractopamine residues in pork

**DOI:** 10.1186/1556-276X-9-639

**Published:** 2014-11-27

**Authors:** Jing Zhang, Xintian Shao, Jingli Yue, Donghui Li, Zhenhua Chen

**Affiliations:** 1 College of Pharmacy, Liaoning Medical University, Jinzhou 121001, People’s Republic of China

**Keywords:** Electrodes, Pork samples, Ractopamine, Nanoparticles

## Abstract

In this work, we reported a simple, fast, and sensitive determination of ractopamine (RAC) residues in pork by using novel ractopamine-tetraphenylborate complexed nanoparticles (RT NPs) as sensors. The prepared RT NPs exhibited a fast response time of 10 s, a wide linear range from 0.1 to 1.0 × 10^−7^ mol/L, and a very low detection limit of 7.4 × 10^−8^ mol/L. The prepared sensor also presents a high selectivity for ractopamine under different pH conditions ranged from 2.85 to 7.18. These results reveal that the fabricated RT NPs can be used as efficient electrochemical sensors to determine ractopamine in animal productions.

## Background

Ractopamine (RAC) is a *β*-agonist and belongs to the phenyl ethanolamine group. Adding RAC to the fodder can reduce the fat deposition and increase the muscle in animals. However, RAC residues in the liver, lung, and other organs will cause a series of health problems for consumers, such as muscular tremors, tachycardia, cardiac palpitation, dizziness, and even death [1]. Because of these potential risks to consumers, RAC is not licensed for animal production in many countries. Therefore, intensive attention has been attracted in developing various analytical methods to assay RAC in animal feeds, tissues, and body fluid, such as HPLC [2-4], gas chromatography–mass spectrometry [5,6], liquid chromatography-mass spectrometry [7-10], fluorescence spectrometry [11,12], and enzyme-linked immunosorbent assay [13-16], and these methods have been reported. However, the overcoming of time-consuming procedures, expensive instrument, complex treatment steps, and the needs of skilled operators are still challenges for convenient and rapid determining of RAC.

**Figure 1:**
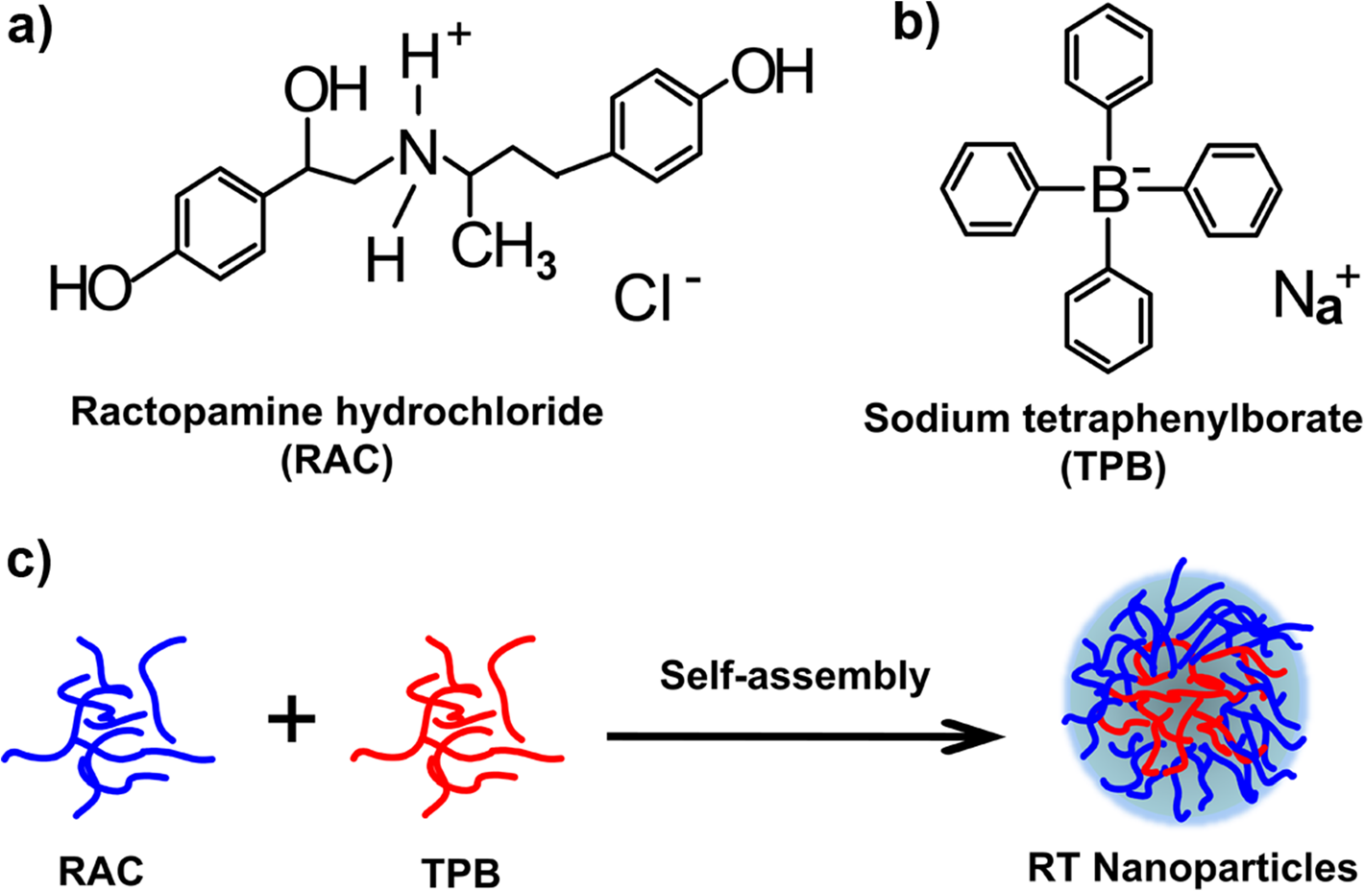
**Chemical structure and schematic illustrations.** Chemical structures of ractopamine **(a)** and sodium tetraphenylborate **(b). (c)** Schematic illustration of RT NP preparation.

In recent years, nanomaterial, as a new class of materials, has been widely applied in various fields, especially in biomedical science [17-21]. Due to their unique physical and chemical properties, such as large surface/volume ratio, excellent electrocatalytic activity, good conductivity, and high mechanical strength, the nanomaterials attracted intensive attention on application of electrochemical sensors and biosensors [17,22]. An electrochemical method has been widely reported in determination of biological samples due to its advantages of low cost, simple operation, and fast detection [23,24]. Therefore, the electrochemical method for determination of ractopamine has attracted much attention of researchers [25-27]. In this paper, as schematic in Figure 1, we proposed a strategy to prepare ractopamine-tetraphenylborate complexed nanoparticles by the virtue of self-assembly induced by the interaction between positive and negative charges. We intend to use the fabricated ractopamine-tetraphenylborate complexed nanoparticles (RT NPs) as modifiers to form an electrochemical sensor with a carbon paste electrode and apply it to determine ractopamine in pork samples conveniently and rapidly.

## Methods

### Reagents

All the chemicals used were analytical grade. Triply distilled deionized water was used during all the experiments. Graphite of high molecular weight, ractopamine hydrochloride (reference standard) and tetraphenylboron sodium (ACS reagent, 99.5%) were purchased from Sigma-Aldrich, St. Louis, MO, USA. The pork samples were purchased from a local supermarket.

### Preparation of RT NPs

The RT NPs were prepared as follows: RAC solution (2.0 mmol/L) was slowly dropped into 2.0 mmol/L tetraphenylboron sodium (TPB) solution under vigorous stirring. Then, the mixture was kept still and aged for 20 h. The obtained precipitates were separated by centrifugation at 18,000 r/min and rinsed by distilled water for five times. Finally, the products were dried at 50°C and stored in dark glass bottles for further characterization and application.

### Preparation of the modified carbon paste electrodes

Bare carbon paste electrodes (CPEs) were prepared by mixing 400 mg of graphite powder and 150 mg solid paraffin with a mortar and pestle. The modified CPEs were prepared in similar procedures, except that 40 mg of RT NPs was added into the graphite powder in an agate mortar. The mixture was put in an incubator until solid paraffin melted completely. Then the paste was immediately packed into a glass tube (1 cm in diameter), and the electrical contact to paste was set up with a copper wire. The electrode surface was smoothed with a slick paper. Before use, the electrode was soaked in 1.0 × 10^−3^ mol/L ractopamine solution for 30 min, to ensure the electrode was activated.

### Sample preparation

The preparation of pork samples follows the method reported in the literature [28-30]. Briefly, 10 g of smashed pork was added into 20 mL 0.1 mol/L HClO_4_. After being homogenized for 2 min, the mixture was centrifuged (at 8,000 rpm, 10 min). After that, the clear liquid was collected and the residue was extracted with the same process again as mentioned above. Both supernatants were merged together, and the pH value was adjusted to 9.0 with 1.0 mol/L NaOH. Then, 3.0 g NaCl was added. Subsequently, 25 mL ethyl acetate was added into the supernatant, and the mixture was vortexed for 10 min. The organic layer was collected and evaporated under N_2_ stream at 60°C. Finally, the extracted product was diluted to 50 mL HAc-NaAc buffer solution. Spiked samples were prepared in the same steps, except a known amount of ractopamine standard were added to the pork sample before treatment.

### Preparation of standard RAC solutions

A stock solution of 0.1 M RAC was prepared. The working solutions (10^−7^ to 10^−1^ M) were prepared by serial appropriate dilution of the stock solution.

### Characterization

To identify the composition of the synthetic products, Fourier transform infrared spectroscopy (FTIR) was performed by using a SHIMADZU spectrum system (SHIMADZU, Kyoto, Japan) with a resolution of 4.00 cm^−1^. The morphologies of the products were studied by scanning electron microscopy (SEM; Hitachi, S4800, Tokyo, Japan) and transmission electron microscopy (TEM; JEM-1200EX, Tokyo, Japan). The mean diameter of the corresponding sample was performed by using dynamic light scattering (DLS; Malvern, Nano ZS90, Worcestershire, UK). The electrochemical data were obtained using a CHI660C electrochemical workstation using cyclic voltammetry and electromotive force measurements. A packed saturated calomel electrode (SCE) was used as an external reference. The typical cell for electrochemical data measurement was assembled as follows:

SCE, KCl (3 M) | sample solution | the general carbon paste electrode or the modified carbon paste electrode.

## Results and discussion

### Characterization of the prepared RT NPs

**Figure 2:**
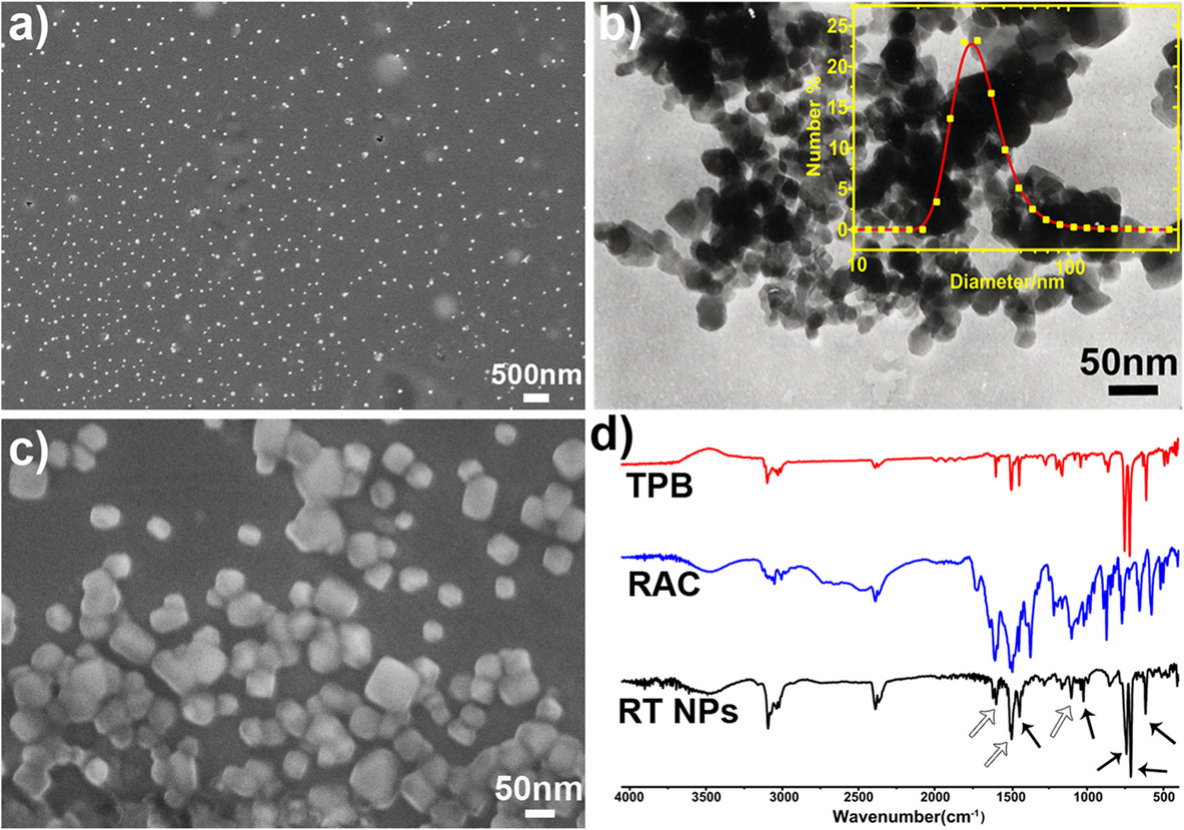
**SEM and TEM images, and FTIR spectra.** SEM images of the obtained RT NPs **(a)** and the surface of the modified electrode **(c)**; TEM image of RT NPs **(b)**; the insert is the size distribution measured by DLS. **(d)** FTIR of RT NPs, RAC, and TPB.

Figure 2a shows the SEM image of the obtained RT NPs; it is clear that the products were monodisperse nanoparticles with sizes of around 50 nm. This was further demonstrated by TEM image and DLS size distribution shown in Figure 2b, and these results confirmed that the size of the prepared RT NPs mainly distributed in the range of 40 to 50 nm. The surface of the carbon paste electrode modified with RT NPs was shown in Figure 2c, and it was obvious that the RT NPs were uniformly scattered on the surface of the electrode. FTIR spectra of RT NPs clearly show the characteristic absorption peaks ascribed to TPB at 612, 707, 735, 1,014, and 1,427 cm^−1^ (Figure 2d, indicated by black arrows) and the typical absorption peaks ascribed to RAC at 1,092, 1,479, and 1,578 cm^−1^ (Figure 2d, indicated by blank arrows). These results suggest the formation of a stable complex between RAC and TPB in RT NPs.

### Sensor properties of RT NPs

**Figure 3:**
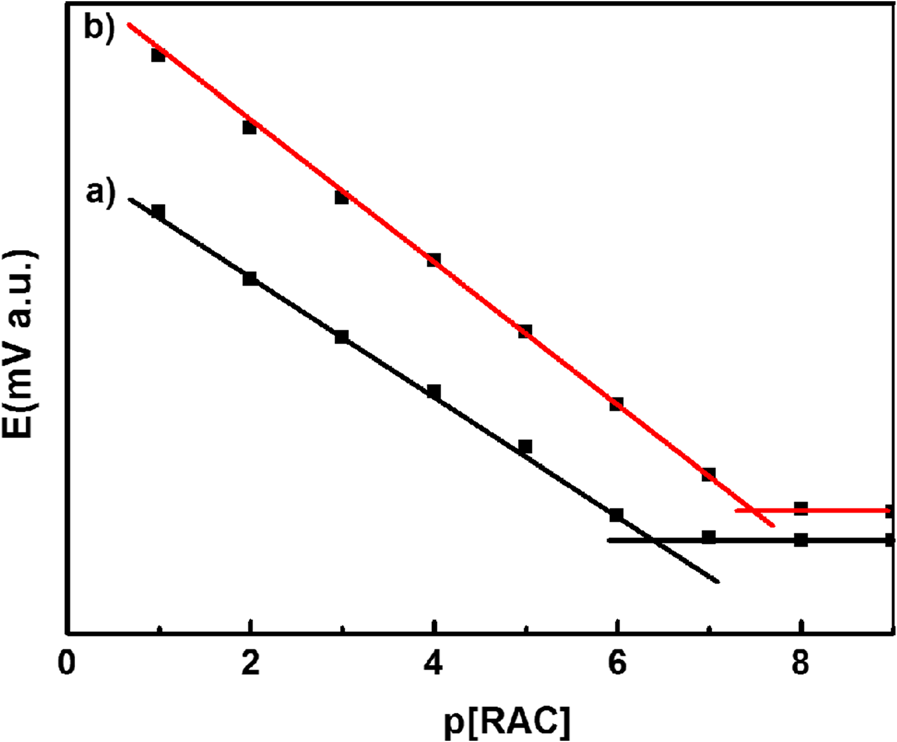
Calibration curves of the general CPEs (a) and the RT NP-modified CPEs (b).

The response range of an ion-selective electrode is the linear part of the calibration curve [17]. As shown in Figure 3, the RT NP-modified CPEs (curve b) presented a wider response range compared to the general CPEs (modified only with RAC, curve a). The results were in line with the Nernstian behavior on the electrodes, and the concentration range is from 0.1 to 1.0 × 10^−7^ mol/L. The detection limit was calculated by the linearization method [17]. Compared to a detection limit of 2.7 × 10^−7^ mol/L for general CPEs to RAC, RT NP-modified CPEs presented a much lower detection limit of 7.4 × 10^−8^ mol/L. Kong et al. reported a detection limit of RAC of 2.38 × 10^−8^ mol/L based on a molecularly imprinted polymer film [25]; Rajkumar and his coworkers reported a detection limit of 1.5 × 10^−7^ mol/L by using zirconia nanoparticle-modified electrodes [26]; and Wu et al. got a detection limit of 17 μg/L by using graphene oxide as sensors [27]. Thus, the detection limit of 7.4 × 10^−8^ mol/L of RT NP-modified CPEs is at a similar detection limit level for RAC reported in the recent literatures.

### Electrochemical behaviors of carbon paste electrodes

**Figure 4:**
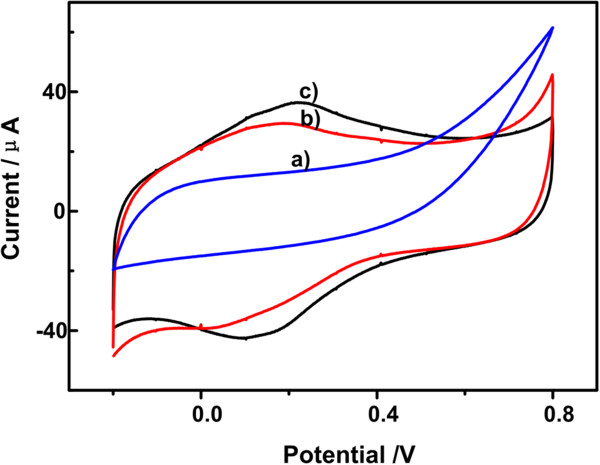
**Cyclic voltammogram curves.** CVs of the bare CPEs **(a)**, the general CPEs **(b)**, and the RT NP-modified CPEs **(c)** in 0.1 mol/L HAc-NaAc buffer containing 1.0 × 10^−3^ mol/L RAC. Scan rate of 100 mV•s^−1^.

Figure 4 shows the cyclic voltammograms (CV) of the bare CPEs (curve a), the general CPEs (curve b), and the RT NP-modified CPEs (curve c) in 0.1 mol/L HAc-NaAc buffer which contains 1.0 × 10^−3^ mol/L ractopamine solution at the scan rate of 100 mV/s. Figure 4a showed no peak current. Figure 4b showed only a small peak current obtained at the general carbon paste electrode. Figure 4c revealed that the redox peak current of the RT NP-modified CPEs increase significantly compared to the other two CPEs. This is most likely due to the presence of RT NPs, which may have increased the surface area of the electrode and provided faster electron transfer between the drug molecules.

### The effect of the scan rate on the oxidation of ractopamine at CPEs

**Figure 5:**
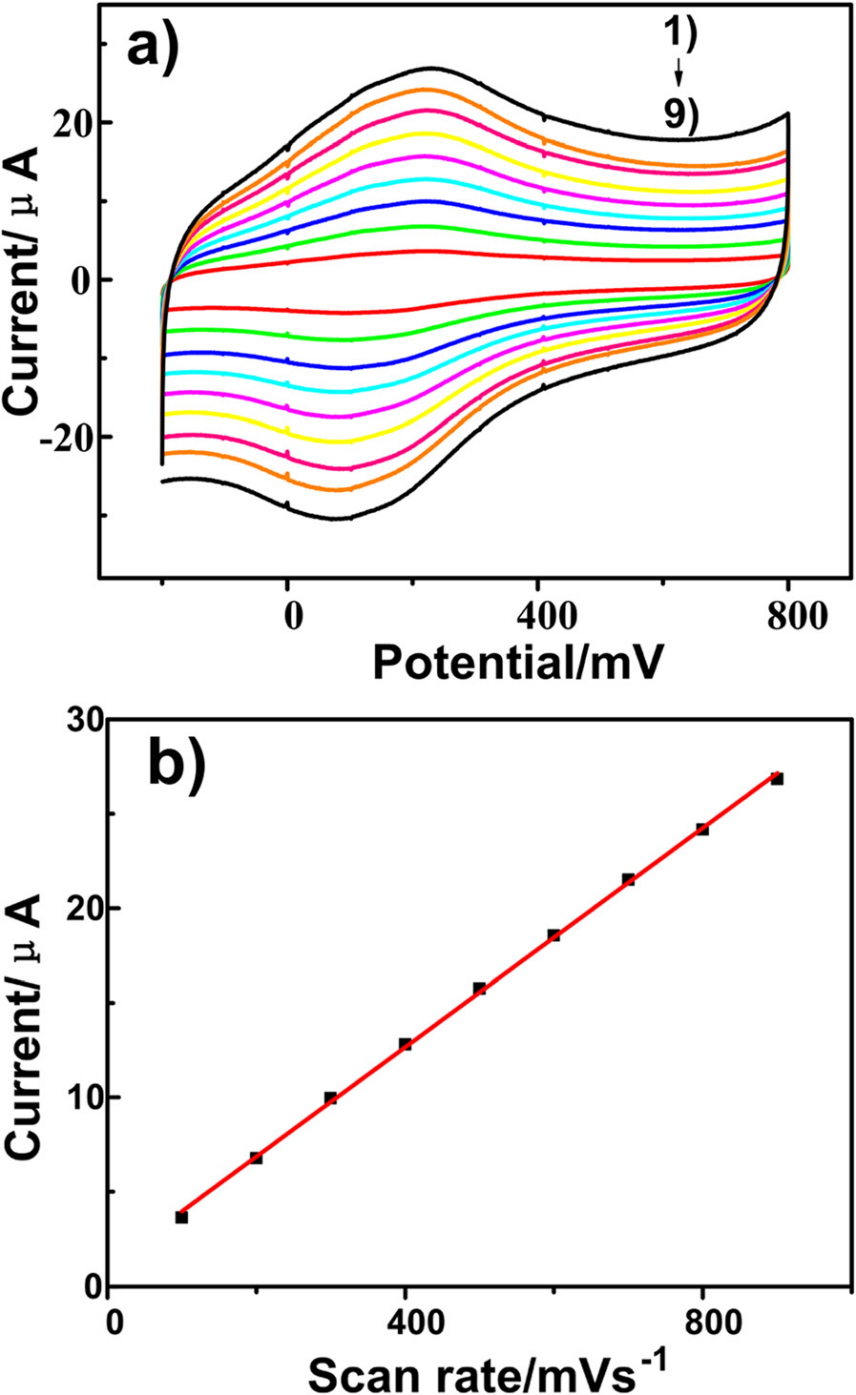
**Cyclic voltammogram curves of the RT NP-modified CPEs at different scan rates. (a)** CVs of the RT NP-modified CPEs in 0.1 M HAc-NaAc buffer containing 1.0 × 10^−3^ mol/L ractopamine at different scan rates (1 to 9): 0.1, 0.2, 0.3, 0.4, 0.5, 0.6, 0.7, 0.8, and 0.9 mV s^−1^. **(b)** Graph of anodic peak current versus scan rate.

Figure 5a shows the cyclic voltammograms of 1.0 × 10^−3^ mol/L RAC at different scan rates in the range of 100 ~ 900 mV/s on the modified CPEs. Figure 5b shows a linear relationship between the peak current and the scan rate. The calibration equation is *I =* 1.06 + 28.98 V and the correlation coefficient is 0.9992. According to the above equation, an adsorption controlled process occurred at the surface of the modified CPEs.

### Response time and lifetime of RT NP-modified CPEs

Response time is an important indicator for the prepared sensor. It refers to the length of time needed by the electrode reaching equilibrium since it soaked into the test solution. The response time was evaluated by changing the concentration of ractopamine from 0.1 to 1.0 × 10^−4^ mol/L. The response time of RT NP-modified CPEs over the concentration range was 10 s, which is much shorter than the response time in general CPEs and the literature result of >100 s [25]. In the low concentration solutions, the response time was calculated as almost 20 s. The lifetime of RT NP-modified CPEs was obtained for 3 months by the normal use, while the general CPEs can be used for 10 weeks. After the lifetime, the properties of the electrode will decrease and the detection limit will increase. Those results are listed in Table 1; the data revealed that the detection limit of the modified CPEs was from 7.4 × 10^−8^ mol/L to 3.4 × 10^−7^ mol/L after 10 weeks of extensive use. This may be because of the loss of exchange of ions in the pastes when soaking CPEs into the solution, which results in the increase of the detection limit and the decrease of the slopes.

**Table 1:** The lifetime of the CPEs

**Week**	**Modified CPEs**		**General CPEs**	
	**Slope**	**Detection limit**	**Slope**	**Detection limit**
First	56	7.4 × 10^−8^	46	2.7 × 10^−7^
Second	55.92	8.0 × 10^−8^	45.87	3.2 × 10^−7^
Third	55.83	8.5 × 10^−8^	45.03	3.8 × 10^−7^
Fourth	55.72	8.9 × 10^−8^	44.74	4.5 × 10^−7^
Fifth	55.43	9.4 × 10^−8^	43.82	6.7 × 10^−7^
Sixth	55.02	9.9 × 10^−8^	42.23	8.1 × 10^−7^
Seventh	54.76	1.2 × 10^−7^	41.36	9.3 × 10^−7^
Eighth	54.28	1.8 × 10^−7^	40.28	1.5 × 10^−6^
Ninth	53.82	2.5 × 10^−7^	39.56	2.3 × 10^−6^
Tenth	53.14	3.4 × 10^−7^	38.94	4.2 × 10^−6^

### Effect of pH on the sensor properties

**Figure 6:**
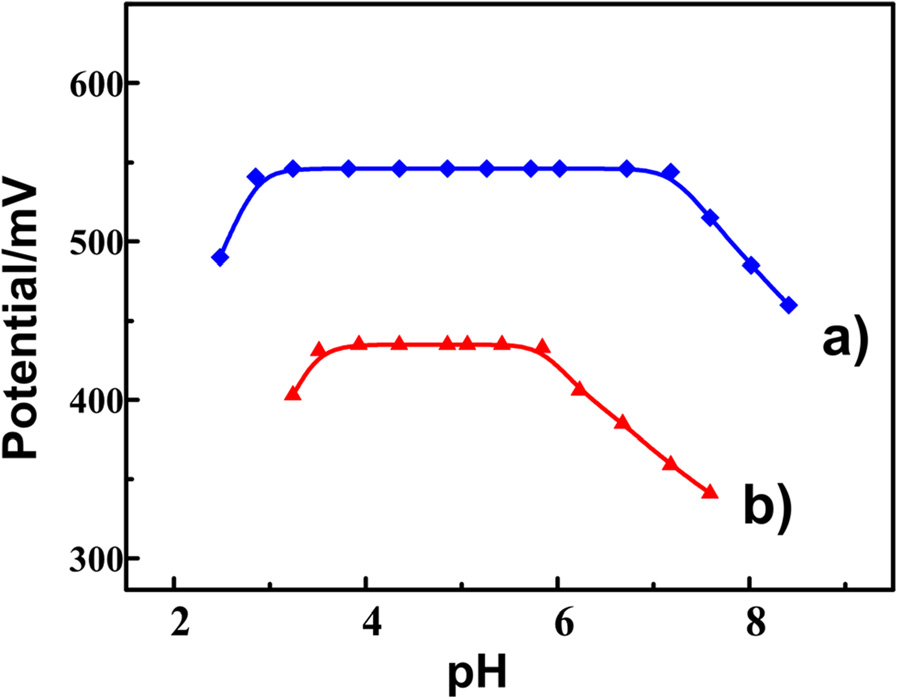
**Effect of pH on the sensor properties.** Effect of pH on **(a)** the modified CPEs and **(b)** the general CPEs in 0.1 M HAc-NaAc buffer containing 1.0 × 10^−3^ mol/L ractopamine.

The value of pH was carried out by a digital pH meter. The effect of pH on the potential values of the RT NP-modified electrodes was investigated in a 1.0 × 10^−3^ mol/L ractopamine solution. The pH adjustments in the solutions were employed by an addition of 0.1 mol/L hydrochloric acid and sodium hydroxide solutions. The effect of pH on CPEs is shown in Figure 6. Figure 6a shows that the response pH range of the RT NP-modified CPEs was from 2.85 to 7.18, which is much wider than that of general CPEs from 3.86 to 5.9 (Figure 6b). It is probably that the RT NPs increased the surface area of the electrode and provided a faster and more stable electron transfer between drug molecules.

### Selectivity of RT NP-modified CPEs

Selectivity is one of the most important characteristics of these electrodes. In order to prove that the prepared electrodes are only responsive to target ions, and other ions are not affected, the interference experiment was carried out. When an ion-selective electrode responds to a major ion, it will be affected by other ions. Thus, selectivity coefficient (*K*_*i,j*_) was used to define the response selectivity of one sensor electrode to target ions, and *K*_*i,j*_ can be calculated by the matched potential method (MPM) from the following equation: *K*_*i,j*_ = *a*_*i*_/*a*_*j*_. The smaller the value of *K*_*i,j*_, the higher the selectivity of the electrode on target ions. Selectivity coefficients of various ions for the RT NP-modified electrode are listed in Table 2. The values of *K*_*i,j*_ were all less than 0.01; those data proved that the prepared RT NP-modified electrode was very selective over the studied interference.

**Table 2:** Selectivity coefficients of various ions for the RT NP-modified electrode

**Interference ions**	***K***_***i,j***_
Cd^2+^	7.05 × 10^−3^
Cr^3+^	2.45 × 10^−3^
Ni^2+^	8.10 × 10^−4^
Zn^2+^	2.55 × 10^−5^
Pb^2+^	8.25 × 10^−4^
Fe^3+^	8.06 × 10^−5^
Hg^2+^	1.26 × 10^−3^
NO_2_^−^	2.90 × 10^−4^
Mn^2+^	1.38 × 10^−4^
Ag^+^	5.41 × 10^−3^
HCO_3_^−^	6.53 × 10^−5^
PO_4_^3−^	6.13 × 10^−6^
Se^4+^	1.05 × 10^−3^
NH_4_^+^	4.67 × 10^−3^

### Determination of ractopamine in pork

In order to verify the proposed RT NP-modified CPEs in practical analysis, the developed method mentioned above was applied for the determination of RAC in pork samples. The pork samples were treated as described before and detected by the electromotive force (EMF) measurement. No RAC was detected in the commercial pork samples; then, the recovery experiments were carried out by the standard addition method. Different concentrations of RAC were added, and the recoveries were measured. The determination results are listed in Table 3.

**Table 3:** **Recovery data obtained for RAC in pork samples using RT NP-modified electrode (*n*** **= 5)**

**Ractopamine added (μM)**	**Ractopamine found (μM)**	**Recovery (%)**	**RSD (%)**
8	8.124 ± 0.293	97.8 ~ 105	1.7
15	15.087 ± 0.193	99.4 ~ 102	1.9
20	20.205 ± 0.284	99.6 ~ 102	0.9
40	40.136 ± 0.257	99.7 ~ 101	1.2

## Conclusions

In this work, the ractopamine-tetraphenylborate complexed nanoparticles were prepared and used as modifiers to fabricate sensor CPEs for ractopamine. An EMF measurement was adopted to determine the ractopamine in pork samples. The results revealed that the good Nernstian response with a detection range of 10^−1^ ~ 10^−7^ mol/L, the lower detection limit of 7.4 × 10^−8^ mol/L, and very rapid response time of 10 s were obtained by the proposed RT NP-modified CPEs. Therefore, the modified electrodes can be used as efficient electrochemical sensors to determine ractopamine in animal productions.

## Competing interests

The authors declare that they have no competing interests.

## Authors’ contributions

JZ and XS did the experiment, data collection, and draft writing; JY gave her contributions on the experimental design and guidance; DL gave his contributions on the experimental design, discussion, and paper modification; and ZC took the contributions on the research design, data analysis, and discussion, as well as the main paper organization. All authors read and approved the final manuscript.

## Authors’ information

ZC got his Ph.D. (major in Biomedical Engineering) from Sichuan University, China. He has focused on biomaterials especially on nanoparticle synthesis and application for almost 10 years. His published papers involved the inorganic and organic nanoparticles toward multifunctional nanocarriers and sensors, and biomineralization.

## References

[B1] Rincker PJ Killefer J Matzat PD Carr SN McKeith FK The effect of ractopamine and intramuscular fat content on sensory attributes of pork from pigs of similar genetics J Muscle Foods 2009 20 79 88 10.1111/j.1745-4573.2008.00135.x

[B2] Freire EF Borges KB Tanimoto H Nogueira RT Bertolini LCT De Gaitani CM Development and validation of a simple method for routine analysis of ractopamine hydrochloride in raw material and feed additives by HPLC J AOAC Int 2009 92 757 764 19610364

[B3] Burnett TJ Rodewald JM Moran J Turberg MP Brunelle SL Coleman MR Determination of ractopamine in swine, bovine, and turkey tissues by HPLC with fluorescence detection: first action 2011.22 J AOAC Int 2012 95 945 958 10.5740/jaoacint.CS2011_22 22970562

[B4] Turberg MP Rodewald JM Coleman MR Determination of ractopamine in monkey plasma and swine serum by high-performance liquid chromatography with electrochemical detection J Chromatogr B Biomed Appl 1996 675 279 285 10.1016/0378-4347(95)00397-5 8852716

[B5] Sniegocki T Zmudzki J Posyniak A Semeniuk S Gas chromatography–mass spectrometric confirmatory method for the determination of clenuterol residues in animal urine and liver samples Bull Vet Inst Pulawy 2003 47 139 144

[B6] He L Su Y Zeng Z Liu Y Huang X Determination of ractopamine and clenbuterol in feeds by gas chromatography–mass spectrometry Anim Feed Sci Tech 2007 132 316 323 10.1016/j.anifeedsci.2006.03.013

[B7] Li C Wu Y-L Yang T Zhang Y Simultaneous determination of clenbuterol, salbutamol and ractopamine in milk by reversed-phase liquid chromatography tandem mass spectrometry with isotope dilution J Chromatogr A 2010 1217 7873 7877 10.1016/j.chroma.2010.10.055 21067758

[B8] Lafontaine C Yu H Espourteille FA Shi Y Quantitative analysis of ractopamine in beef using automated online sample preparation with liquid chromatography-tandem mass spectrometry Anal Methods 2012 4 3536 3541 10.1039/c2ay25497b

[B9] Thevis M Schebalkin T Thomas A Schänzer W Quantification of clenbuterol in human plasma and urine by liquid chromatography-tandem mass spectrometry Chromatogr 2005 62 435 439 10.1365/s10337-005-0651-3

[B10] Blanca J Muñoz P Morgado M Méndez N Aranda A Reuvers T Hooghuis H Determination of clenbuterol, ractopamine and zilpaterol in liver and urine by liquid chromatography tandem mass spectrometry Anal Chim Acta 2005 529 199 205 10.1016/j.aca.2004.09.061

[B11] Ni Y Wang Y Kokot S Voltammetric, UV–vis spectrometric and fluorescence study of the interaction of ractopamine and DNA with the aid of multivariate curve resolution‒alternating least squares Electroanal 2010 22 2216 2224 10.1002/elan.200900596

[B12] Ni Y Zhang Q Kokot S Analysis of the interactions of mixtures of two β-agonists steroids with bovine serum albumin: a fluorescence spectroscopy and chemometrics investigation Analyst 2010 135 2059 2068 10.1039/c0an00161a 20544093

[B13] Shelver WL Kim HJ Li QX Development of a monoclonal antibody-based enzyme-linked immunosorbent assay for the β-adrenergic agonist zilpaterol J Agr Food Chem 2005 53 3273 3280 10.1021/jf0477954 15853359

[B14] Lei YC Tsai YF Tai YT Lin CY Hsieh KH Chang TH Kuo TF Development and fast screening of salbutamol residues in swine serum by an enzyme-linked immunosorbent assay in Taiwan J Agr Food Chem 2008 56 5494 5499 10.1021/jf800625f 18578536

[B15] Shelver WL Smith DJ Enzyme-linked immunosorbent assay development for the β-adrenergic agonist zilpaterol J Agr Food Chem 2004 52 2159 2166 10.1021/jf049919i 15080615

[B16] Pleadin J Perši N Vulić A Milić D Vahčić N Determination of residual ractopamine concentrations by enzyme immunoassay in treated pig's tissues on days after withdrawal Meat Sci 2012 90 755 758 10.1016/j.meatsci.2011.11.007 22119670

[B17] Yue J-L Chen Z-H E Y-F Chen L-S Zhang J Song Y-M Zhai Y-C Preparation TiO2 core-shell nanospheres and application as efficiency drug detection sensor Nanoscale Res Lett 2014 9 1 6 10.1186/1556-276X-9-1 25246870 PMC4158343

[B18] Chen Z Wang C Chen J Li X Biocompatible, functional spheres based on oxidative coupling assembly of green tea polyphenols J Am Chem Soc 2013 135 11 4179 4182 10.1021/ja311374b 23470166

[B19] Wang J Xu D Kawde AN Polsky R Metal nanoparticle-based electrochemical stripping potentiometric detection of DNA hybridization Anal Chem 2001 73 5576 5581 10.1021/ac0107148 11816590

[B20] Zhu Z Su Y Li J Li D Zhang J Song S Fan C Highly sensitive electrochemical sensor for mercury (II) ions by using a mercury-specific oligonucleotide probe and gold nanoparticle-based amplification Anal Chem 2009 81 7660 7666 10.1021/ac9010809 19691296

[B21] Guo S Wen D Zhai Y Dong S Wang E Platinum nanoparticle ensemble-on-graphene hybrid nanosheet: one-pot, rapid synthesis, and used as new electrode material for electrochemical sensing ACS Nano 2010 4 3959 3968 10.1021/nn100852h 20568706

[B22] Mashhadizadeh MH Afshar E Electrochemical investigation of clozapine at TiO_2_ nanoparticles modified carbon paste electrode and simultaneous adsorptive voltammetric determination of two antipsychotic drugs Electrochim Acta 2013 87 816 823

[B23] Ikebukuro K Kiyohara C Sode K Novel electrochemical sensor system for protein using the aptamers in sandwich manner Biosens Bioelectron 2005 20 2168 2172 10.1016/j.bios.2004.09.002 15741093

[B24] Hu Y Mitchell KM Albahadily FN Michaelis EK Wilson GS Direct measurement of glutamate release in the brain using a dual enzyme-based electrochemical sensor Brain Res 1994 659 117 125 10.1016/0006-8993(94)90870-2 7820652

[B25] Kong LJ Pan MF Fang GZ Qian K Wang S An electrochemical sensor for rapid determination of ractopamine based on a molecularly imprinted electrosynthesized o-aminothiophenol film Anal Bioanal Chem 2012 404 1653 1660 10.1007/s00216-012-6253-7 22820950

[B26] Rajkumar M Li YS Chen SM Electrochemical detection of toxic ractopamine and salbutamol in pig meat and human urine samples by using poly taurine/zirconia nanoparticles modified electrodes Colloids Surf B Biointerfaces 2013 110 242 247 23732800 10.1016/j.colsurfb.2013.03.038

[B27] Wu C Sun D Li Q Wu K Electrochemical sensor for toxic ractopamine and clenbuterol based on the enhancement effect of graphene oxide Sensors Actuat B Chem 2012 168 178 184

[B28] Liu Z Zhou Y Wang Y Cheng Q Wu K Enhanced oxidation and detection of toxic ractopamine using carbon nanotube film-modified electrode Electrochim Acta 2012 74 139 144

[B29] Yang X Feng B Yang P Ding Y Chen Y Fei J Electrochemical determination of toxic ractopamine at an ordered mesoporous carbon modified electrode Food Chem 2014 145 619 624 24128523 10.1016/j.foodchem.2013.08.093

[B30] Lu X Zheng H Li X-Q Yuan X-X Li H Deng L-G Aboul-Enein HY Detection of ractopamine residues in pork by surface plasmon resonance-based biosensor inhibition immunoassay Food Chem 2012 130 1061 1065 10.1016/j.foodchem.2011.07.133

